# Gut Microbiota in Neurological Disorders

**DOI:** 10.1007/s00005-019-00561-6

**Published:** 2019-10-01

**Authors:** Marta Grochowska, Tomasz Laskus, Marek Radkowski

**Affiliations:** 1 Department of Immunopathology of Infectious and Parasitic Diseases, Medical University of Warsaw, Warsaw, Poland; 2 Department of Adult Infectious Diseases, Medical University of Warsaw, Warsaw, Poland

**Keywords:** Gut, Microbiota, Neurological disorders

## Abstract

The incidence of neurological disorders such as multiple sclerosis (MS), Alzheimer’s disease (AD) and Parkinson’s disease (PD) is increasing throughout the world, but their pathogenesis remains unclear and successful treatment remains elusive. Bidirectional communications between the central nervous system and gut microbiota may play some role in the pathogenesis of the above disorders. Up to a thousand bacterial species reside in human intestine; they colonize the gut shortly after birth and remain for life. Numerous studies point to the role of microbiota composition in the development, course and treatment of MS, AD and PD.

## Introduction

The purpose of this review is to summarize the current knowledge about the role of gut microbiota composition in the development, course, and prognosis of selected neurological diseases. Global incidence of a number of common neurological disorders is rapidly increasing (Bengmark 2013) and consumption of industrially manipulated meat, dairy and wheat products, which lead to changes in gut microbiota, could be contributing factors (Bengmark 2007). Understanding the effects of diet on human gut microbiota is therefore of major importance (Kau et al. 2011).

The central nervous system (CNS) and the gut microbiota interact in a bidirectional manner, e.g. the CNS modulates gut functioning in response to psychological and physical stressors affecting motility, secretion and immune reactivity (Mayer 2000), whereas changes in gut microbiota may cause behavioural and neurochemical changes (Bravo et al. 2011). Consequently, the term microbiota–gut–brain (MGB) axis was proposed to stress the importance of these interactions (Collins et al. 2012; Rhee et al. 2009). the MGB regulates the gastrointestinal (GI) tract and CNS (Dinan et al. 2014) by way of the vagus nerve, hypothalamic–pituitary–adrenal axis and various cytokines (Dinan et al. 2014; Jones et al. 2006).

Up to a thousand bacterial species live in human intestine (Mazmanian et al. 2008), and as many as 10^11^ bacteria cells can be found in 1 g of colon contents (Guarner and Malagelada 2003). This particular ecosystem includes not only bacteria, which are often unculturable (Xu et al. 2007), but also viruses, protozoa, archae and fungi (Dinan and Cryan 2012). Commensal bacteria colonize the human intestine shortly after birth and remain there for life (Foster and McVey Neufeld 2013). The most common phyla are *Firmicutes* and *Bacteroidetes*, which represent 70–75% of total microbiome defined as genetic material of all intestinal microbes. Other bacteria occurring in lower quantities are *Proteobacteria, Actinobacteria, Fusobacteria* and *Verrucomicrobia* (Eckburg et al. 2005). The microbiome composition is affected by diet, metabolism, age, geography, stress and antibiotic treatment (Foster and McVey Neufeld 2013). For example *Bacteroides spp.* dominate in people on long-term protein and animal fat diet, whereas *Prevotella spp.* dominate in those on carbohydrate diet (Wu et al. 2011).

Intestinal microbiota can modulate gut functioning, e.g. *Bifidobacterium bifidum* and *Lactobacillus acidophilus* promote motility (Rhee et al. 2009), whereas *Escherichia* species exert the opposite effect (Mazmanian et al. 2008; Rhee et al. 2009). Gut dysbiosis, which is defined as a major shift in microbial composition and function (Fond et al. 2015) can cause diarrhea or constipation (Rhee et al. 2009), but even small changes in the gut microbiota can provoke clinical symptoms (Bravo et al. 2012). Overall, microbiota composition is highly individualized and dynamic (Foster and McVey Neufeld 2013).

Intestinal mucosa, which represents a barrier between the internal and external environments, is subjected to stress (Haq et al. 2019). This barrier function is supported by diverse bacteria forming commensal microflora (Brown 2016). The intestinal mucosa is responsible for absorption of water, nutrients and gases, clearance of waste, maintenance of immunity and much more (Haussner et al. 2019). Intestinal mucosa, gut microbiota, immune cells in the mucosa and various products of epithelial origin are all components of the so called “gut barrier”. If the integrity of the latter becomes compromised, leaky gut syndrome (LGS) ensues leading to systemic- and neuroinflammation and causing dysfunction in the cerebellum and hippocampus (Bengmark 2013; Daulatzai 2014; Farhadi et al. 2003). Importantly, patients with many CNS disorders were found to have increased intestinal permeability (Maes et al. 2012), and passing of harmful metabolites from intestine to blood can negatively affect the CNS (Julio-Pieper et al. 2014). Due to the damaged intestinal barrier, lipopolysaccharides (LPS), which are large molecules found in the outer membrane of Gram-negative bacteria, or even whole bacteria (Bengmark 2013), may enter the bloodstream causing endotoxemia and activating the immune system (Bengmark 2013). Intestinal permeability can be assessed by functional tests (sucrose test and cellobiose plus mannitol test), serological tests (antibodies to food antigens, autoantibodies), as well as by other markers (e.g. Zonulin serum levels) (Julio-Pieper et al. 2014).

While dysfunction in microbiota could play a role in the development of some neurological diseases, there is also mounting evidence that interventions restoring its health and intestinal barrier integrity can positively affect clinical course and symptoms (Julio-Pieper et al. 2014). The most common treatments targeting gut dysbiosis are probiotics, which are defined as living organisms providing health benefits to the host (Bravo et al. 2012), and antibiotics.

## Multiple Sclerosis

Multiple sclerosis (MS) is the most common neurological disease of young adults in Europe and North America (Keegan and Noseworthy 2002). It is characterized by an autoimmune inflammatory process in which CD4^+^CD25^+^ regulatory T (Treg) cells target brain and spinal cord cells leading to demyelination and axonal damage (Lavasani et al. 2010; Tremlett et al. 2016b).

A combination of genetic and environmental factors is likely to be involved in MS pathogenesis (Granieri et al. 2000), but none has been found to be essential (Tremlett and Waubant 2017). There is mounting evidence that an important role could be played by changes in the gut microbiome (Adamczyk-Sowa et al. 2017). While the specific mechanisms have not been elucidated as yet (Forbes et al. 2016), it seems that gut microbiota changes can cause a pro-inflammatory state resulting in CNS damage, the culmination of which is the development of MS (Adamczyk-Sowa et al. 2017; Rodriguez et al. 2016).

Bacteria species likely to be involved are *Helicobacter, Clostridium* and *Enterococcus* (Round and Mazmanian 2009). It was also shown that segmented filamentous bacteria can activate intestinal Th17 cells, which promote systemic autoimmunity and participate in immune response against intestinal pathogens (Atarashi et al. 2011). However, some findings point also to the ability of specific bacteria to provide anti-inflammatory effects (Adamczyk-Sowa et al. 2017). For example, *Bacteroides fragilis’* polysaccharide product was found to be able to modulate systemic Th1 and mucosal Treg cell responses in mice (Mazmanian et al. 2008).

Compared to healthy controls, MS patients have a decrease in the proportion of *Faecalibacterium, Eubacterium rectale, Corynebacterium, Fusobacteria* and an increase of *Escherichia, Shigella, Clostridium, Firmicutes* (Cantarel et al. 2015; Tremlett et al. 2016a, c). Decreased numbers of *Faecalibacterium* spp. and lower levels of its metabolite butyrate lead to decrease of Treg cells, antigen-presenting cells, and pro-inflammatory cytokines (Adamczyk-Sowa et al. 2017; Machiels et al. 2014). Several authors reported a decrease of such *Bacteroides* spp. as *Bacteroides stercoris and Bacteroides coprocola* in the gut microbiota of MS patients and a negative correlation between the number of *Prevotella copri* and the risk of MS development (Miyake et al. 2015).

Tremlett et al. (2016b) found no differences in immune markers such as the number of Th2, Th17 and Tregs cells between children with MS and healthy controls. However, abundance of gut microbiota correlated positively with Th17 in affected children, but not in controls, whereas *Bacteroidetes* spp. numbers correlated inversely with Th17. The abundance of *Fusobacteria* correlated positively with Tregs only in controls.

Some bacterial products could play a role in the communication between the gut and brain. For example, serum levels of *Bactroidetes* spp. metabolite lipid 654 were found to be significantly lower in MS patients than in healthy controls, and are even regarded by some as an MS biomarker (Farrokhi et al. 2013; Kleinewietfeld et al. 2013). Another example is *Clostridium perfringens* toxins B and D (Uzal et al. 2004), which are able to induce such symptoms as lack of movement coordination or blurred vision, which are similar to MS (Adamczyk-Sowa et al. 2017). In MS patients these toxins may cause inflammation of the retina by affecting the barrier veins or by binding to vascular system receptors (Barnett et al. 2009; Fennessey et al. 2012; Nagahama and Sakurai 1992). Toxins of *C. perfringens* are not detectable in healthy subjects (Adamczyk-Sowa et al. 2017; Murrell et al. 1986; Rumah et al. 2013).

Studies on the role of gut microbiota in experimental autoimmune encephalomyelitis (EAE), which is the most widely used animal model of MS, showed that oral treatment with ampicillin, vancomycin, neomycin, sulfate, and metronidazole can delay the onset and reduce the severity of the disease, decrease levels of pro-inflammatory cytokines and increase levels of interleukin (IL)-10 and IL-13 (Ochoa-Reparaz et al. 2009).

Administration of probiotics (IRT5 containing *Lactobacillus casei, Lactobacillus acidophilus, Lactobacillus reuteni, Bifidobacterium bifidum,* and *Streptococcus thermophilus*) before the induction of EAE resulted in its delayed onset and milder course (Forbes et al. 2016; Kwon et al. 2013). In another experiment, treatment by probiotics containing *Lactobacillus paracasei* and *L. plantarum* suppressed the development of EAE and reduced severity of clinical symptoms while increasing the number of Treg cells in mesenteric lymph nodes, and increasing production of transforming growth factor β1 and IL-27. Interestingly, this effect was not observed when the two bacteria species were administered separately (Forbes et al. 2016; Lavasani et al. 2010).

The effects of microbiota therapeutic interventions in humans have not been studied extensively. However, MS patients who underwent fecal microbiota transplantation showed amelioration of neurologic symptoms and improvement of life quality (Evrensel and Ceylan 2016), but these studies were too small to be conclusive. It should be stressed that unhealthy life style choices such as high consumption of salt, animal fat, carbohydrates and low physical activity negatively affect gut microbiota composition and metabolism leading to LGS and exacerbation of MS symptoms (Adamczyk-Sowa et al. 2017; Riccio and Rossano 2015).

## Alzheimer’s Disease

Alzheimer’s disease (AD) is a common neurodegenerative disease of the elderly accounting for 60–80% of all dementia cases (Alzheimer’s Association 2015; Hu et al. 2016). It is expected that the number of patients with dementia worldwide will double every 20 years reaching 115 millions in 2050 (Prince et al. 2013). AD is characterized by the accumulation in the cerebral cortex and other brain regions of misfolded amyloid-β (Aβ) fibrils and oligomers, such as neurofibrillary tangles consisting of hyperphosphorylated tau protein (Mager et al. 2014). There is also evidence for the presence of neuroinflammation and prion-like pathology (Scheperjans 2016a), which are being intensively studied to identify potential causes (Tremlett et al. 2017). It is currently believed that both environmental and genetic factors are involved in the pathogenesis of AD (Hu et al. 2016).

Clearly, some role in the pathogenesis could be played by LGS, which causes increased permeability of blood–brain barrier (BBB), Aβ accumulation in brain and inflammatory response (Hu et al. 2016). LPS, which elicit strong immune responses (Bengmark 2013) were found to be elevated in AD patients (Zhang et al. 2009), while in mice models LPS increases Aβ levels in the brain (Jaeger et al. 2009) and causes severe memory loss (Kahn et al. 2012).

High-fat and/or high-calorie diet seems to be a risk factor for AD development (Eskelinen et al. 2008; Knight et al. 2014). Diet can affect gut microbiota in multiple ways; for example gluten and sugars stimulate systemic inflammation and consequently affect CNS functions (Hu et al. 2016). Such components of diet as deep-sea fish, nuts, and vegetable oils (Gu et al. 2010), which are all major dietary sources of Omega 3 polyunsaturated fatty acids (ω-3 PUFAs) can lower the risk of AD (Morris et al. 2003), and AD patients often demonstrate low levels of DHA (docosahexaenoic acid), which is one of the ω-3 PUFAs. Importantly, gut microbiota is involved in the absorption and metabolism of ω-3 PUFAs (Hu et al. 2016; Tully et al. 2003).

PUFAs include such important compounds as essential fatty acids (EFA), which are not synthetized by humans (Yehuda et al. 2005). EFA affect brain function and seem to be important for many brain disorders (Yehuda et al. 2005). Since EFA and PUFAs must be derived from food, they are capable of crossing the BBB. Decreased brain levels of EFA, especially in the cortex and hippocampus, which mediate learning and memory, were reported in AD patients, and this deficiency was likely due to the reduction in their ability to cross the BBB (Yehuda et al. 2005). Decreased brain EFA levels are likely to play a role in cognitive decline (Yehuda et al. 2002).

Fruits and vegetables lower the risk of AD probably by providing antioxidants and vitamins (Hughes et al. 2010). The same effect could be conferred by antioxidant polyphenols found in coffee, which reduce brain injury caused by oxidative stress (Hu et al. 2016). Accordingly, it was reported that drinking three to five cups daily decreases the risk of AD by 65% compared to drinking less than two cups per day (Eskelinen et al. 2009). Diet and specific nutrients seem to affect gut microbiota composition and influence the production and aggregation of amyloid proteins (Friedland 2015; Scott et al. 2013).

Amyloid-β accumulation is the cornerstone feature of AD, but the exact mechanisms behind this phenomenon are not fully understood (Cattaneo et al. 2017). Cognitively impaired patients with brain Aβ deposition were reported to have lower stool abundance of anti-inflammatory *E. rectale* and *B. fragilis* and higher abundance of pro-inflammatory *Escherichia* spp. and S*higella* spp. when compared to cognitively impaired patients with no Aβ deposition or healthy controls (Cattaneo et al. 2017; Mancuso and Santangelo 2018). These differences could be reflected in the inflammatory state, as the first group was reported to have higher levels of pro-inflammatory cytokines (IL-6, CXCL2, NLRP3, and IL-1β) and lower levels of anti-inflammatory IL-10 (Cattaneo et al. 2017).

*Lactobacillus* spp. and *Bifidobacterium* spp. can produce gamma-Aminobutyric acid (GABA) (Barrett et al. 2012) and may improve cognitive functioning of AD patients when used as probiotics. *Post mortem* studies of AD brains showed reduced GABA concentrations in many cortical and limbic regions (cingulate, amygdala and thalamus) (Lanctot et al. 2004).

Cognitive decline is the key feature of AD (Archer et al. 2011; Carlino et al. 2013) and it has been found that it impairs life quality much more than other behavioural or even psychotic symptoms (Nuechterlein et al. 2011). Cognitive disturbances may be associated with altered activity of various growth factors, including neurotrophins such as brain-derived neurotrophic factor (BDNF) (Carlino et al. 2013). BDNF is involved in such processes as neuronal development and differentiation, as well as synaptic plasticity (Carlino et al. 2013) and its expression may be modulated by gut microbiota: it was reported that specific pathogen-free mice have higher BDNF mRNA levels in the hippocampus and amygdala compared to germ-free animals (Diaz Heijtz et al. 2011). *Post mortem* studies of AD brains showed a 30% decrease in the amount of proBDNF compared to controls and this decrease was as high as 40% in end-stage patients (Michalski and Fahnestock 2003). BDNF serum levels in AD patients are lower than in subjects with other types of dementia or healthy controls (Carlino et al. 2013; Nieto et al. 2013). However, while serum levels of BDNF are easy measured, they are neither predictive of AD development nor correlate with Functional Assessment Staging (Laske et al. 2006; Yasutake et al. 2006).

There is some evidence that bacteria and bacterial endotoxins may be directly involved in the pathogenesis of AD (Asti and Gioglio 2014). Thus, it was reported that infusion of LPS into the fourth ventricle in rats causes brain changes similar to those found in AD patients (Asti and Gioglio 2014). In human studies, transcriptionally active *Chlamydia pneumoniae* was detected in autopsy brains tissue of AD patients (Balin et al. 1998) and Spirochaetes (*Borrelia burgdorferi, Treponema pallidum*) were found in cerebrospinal fluid and the cerebral cortex of patients with general paresis suggesting that these bacteria might play a role in slowly progressive dementia, cortical atrophy, and local amyloidosis (Asti and Gioglio 2014; Miklossy 2011). Furthermore, serum IgG and IgA against *Helicobacter pylori* were reported to be more common in AD patients (Malaguarnera et al. 2004), while *Escherichia coli* endotoxin may facilitate formation of Aβ fibrils in vitro (Asti and Gioglio 2014).

The gut microbiota may influence AD in various ways. Most notably, paucity of gut bacteria may contribute to the development of this disease as a negative correlation was observed between the incidence of AD and microbiota diversity. In the developed countries with high level of hygiene and consequently decreased microbial diversity, the incidence of AD is increasing (Fox et al. 2013). Also bacterial products such as amyloids and LPS, may accumulate in the blood and brain and contribute to the pathological features of AD (Zhao and Lukiw 2015). Some bacteria species, e.g. *Firmicutes, Bacteroidetes*, *Proteobacteria,* produce amyloids inducing pro-inflammatory IL-17A and IL-22 cytokines, which are linked to chronic inflammatory diseases, including AD (Hill and Lukiw 2015; Zhang et al. 2013). Further, gut microbiota changes may cause activation of pro-inflammatory cytokines and consequently increase intestinal permeability (Bekkering et al. 2013), especially in forms of AD characterized by pronounced learning problems and memory loss (Hu et al. 2016). Finally, the gut microbiota is responsible for the production of such vitamins as B12, which reduces the risk of dementia (Hu et al. 2016) and AD (Quadri et al. 2004).

Interestingly, administration of *Bifidobacterium lactis* HN019 decreases the levels of pro-inflammatory cytokines IL-5, IL-6, IL-1β, IL-8 and tumor necrosis factor α (Rincon et al. 2014; Wang et al. 2015). It was also shown that probiotics are able to increase the levels of anti-inflammatory cells (total, helper CD4^+^, and activated CD4^+^CD25^+^ T cells in the peripheral circulation) (Gill et al. 2001; Pistollato et al. 2016).

## Parkinson’s Disease

The mean time from diagnosis to death in Parkinson’s disease (PD), the second most common neurodegenerative disorder of elderly, is 15 years (Forsyth et al. 2011; Katzenschlager et al. 2008; Lees et al. 2009). PD prevalence is approximately 0.4% and the number of cases is predicted to double by the year 2040 (Adams-Carr et al. 2016; Kowal et al. 2013). The median age for the onset of PD is 60 years and the ensuing disabilities are characterized by motor and non-motor symptoms including dementia, which is life-threatening condition (Hobson et al. 2010; Marras and Lang 2008). The majority of PD cases are diagnosed on the basis of neurological symptoms such as tremor, bradykinesia, rigidity, when a significant proportion of neurons within the substantia nigra is already damaged (Adams-Carr et al. 2016; Forsyth et al. 2011).

The key feature of PD is the presence of neuronal inclusions (Lewy Bodies or Lewy neurites) consisting of aggregated and phosphorylated α-synuclein (Braak and Del Tredici 2008). While the cause of PD remains unknown, the GI tract is the source of many toxins affecting the CNS (Julio-Pieper et al. 2014) and interacts with the brain by the dorsal motor nucleus of the vagus nerve. The latter nucleus seems to be the earliest site of Lewy Bodies expression in the course of the disease (Braak and Del Tredici 2008; Forsyth et al. 2011). Several studies reported on the presence of Lewy Bodies in the esophagus and colon (Kupsky et al. 1987; Lebouvier et al. 2008, 2010; Qualman et al. 1984), which suggests that GI tract is somehow involved in the pathogenesis of PD.

Non-motor symptoms, including mood and cognitive impairment, sleep disorders, sensory disruption and GI dysfunction (Postuma et al. 2012), are common in PD and can severely impact patients’ quality of life (Gallagher et al. 2010). The second most common non-motor symptom after anosmia is constipation affecting 50% of patients. It is also a strong risk factor for PD (Adams-Carr et al. 2016). Moreover, functional and structural changes in GI tissues, such as α-synuclein accumulation in the enteric nervous system (ENS) have been described in PD patients (Visanji et al. 2015). The ENS is responsible for normal gut functioning and represents a way by which commensal bacteria regulate many physiological functions, including intestinal motility (Kunze et al. 2009).

Gut microbiota abnormalities are common in PD patients and their relation to brain dysfunction is being intensively investigated (Scheperjans 2016b). In three different studies of fecal microbiota composition there was a decreased representation of bacteria *Prevotellaceae, Coprococcus, Firmicutes, Clostridium coccoides, Clostridium leptum, B. fragilis* and increased representation of *Lactobacillaceae, Verrucomicrobiaceae, Ruminococcaceae, Lactobacuillus, Bacteroidetes, Proteobacteria, Clostridiaceae* and *Akkermansia* (Hasegawa et al. 2015; Keshavarzian et al. 2015; Scheperjans et al. 2015). The increase in abundance of *Lactobacillaceae* could affect the ENS as these bacteria were found to increase the excitability of colonic after-hyperpolarization neurons by inhibiting calcium-dependent potassium channel opening (Kunze et al. 2009). *Lactobacillus* spp. quantity and serum leptin concentrations were found to be positively correlated (Queipo-Ortuno et al. 2013) while the decreased abundance of *Prevotellaceae* and increased abundance of *Lactobacillaceae* have been correlated with decreased levels of gut ghrelin. The latter hormone may regulate nigrostriatal dopamine function and restrict neurodegeneration in PD (Queipo-Ortuno et al. 2013; Scheperjans et al. 2015) and its secretion is decreased in PD patients (Unger et al. 2011). Although the diminished *Prevotellaceae* levels are not specific for PD and were reported in other diseases such as autistic spectrum disorders (Kang et al. 2013) or type 1 diabetes (Brown et al. 2011), increased fecal *Prevotellaceae* levels have been proposed as an excluding biomarker of PD (Scheperjans et al. 2015). Thus, the quantity of these bacteria was 77.6% lower in PD patients when compared to sex- and age-matched controls. Relative abundance of *Prevotellaceae* below 6.5% was found to be diagnostic of PD with 86.1% sensitivity and 38.9% specificity (Dinan and Cryan 2017). It was also reported that high levels of *Enterobacteriaceae* positively correlate with the severity of postural instability and gait difficulty (Scheperjans et al. 2015) supporting the existence of a link between gut microbiota composition and motor disturbances in PD.

Initial studies employing per os administration of hydrogen water in rats (Fu et al. 2009) and mice (Fujita et al. 2009) demonstrated its protective role in animal models of PD and a subsequent double-blind, randomized, controlled study in humans showed that 1000 ml of hydrogen water per day improved the total Unified Parkinson’s Disease Rating Scale scores (Yoritaka et al. 2013). Consequently, the hydrogen-producing bacteria were analyzed in PD patients. There was a decrease of six bacterial groups producing hydrogen: *B*. *fragilis*, *C*. *perfringens*, *Pseudomonas spp.,* most strains in *Enterobacteriaceae* family, 12 species in *Clostridium*, *C*. *coccoides* group and *C*. *leptum* subgroup (Hasegawa et al. 2015).

Interestingly, small intestinal bacterial overgrowth was found to contribute to the pathophysiology of motor fluctuation in PD patients and eradication of bacterial overgrowth resulted in clinical improvement (Fasano et al. 2013), and it was also reported that patients with irritable bowel syndrome have an increased risk of developing PD (Lai et al. 2014). It has been proposed that gut microbiota in PD patients are shifted towards pro-inflammatory state, since certain genes related to the production of LPS and “pro-inflammatory” bacterial taxes like *Akkermansia* and *Ralstonia* were found to be increased in affected patients (Scheperjans 2016b).

Some authors also found an increase in gut permeability in patients with PD when using the urinary sucralose test (Julio-Pieper et al. 2014). This could results in passing of harmful substances into circulation (Forsyth et al. 2011).

## Conclusions

There was a 90% decrease of plant fiber intake between 1800 and 1970, while the consumption of calories from animal fat and refined sugar increased four times in this time period (Burkitt et al. 1972). As the concentration of plant fiber in our diet fell below 20% (Bengmark 2013), the GI transit time increased five-fold (Burkitt et al. 1972). These Western dietary habits caused a decrease both in absolute number and diversity of gut bacteria (Bengmark 2013).

There is ample evidence for the existence of communication between CNS, gut and intestinal microbiome (Rhee et al. 2009) and analysis of relationships between gut microbiota composition and CNS disorders has become a novel and promising field of research, which could provide understanding of disease pathogenesis and offer new and effective treatment options (Table 1) (Julio-Pieper et al. 2014; Luna and Foster 2015; Scheperjans 2016b). Future clinical studies of microbiota manipulations should be based on randomised, controlled trials.

**Table 1:**
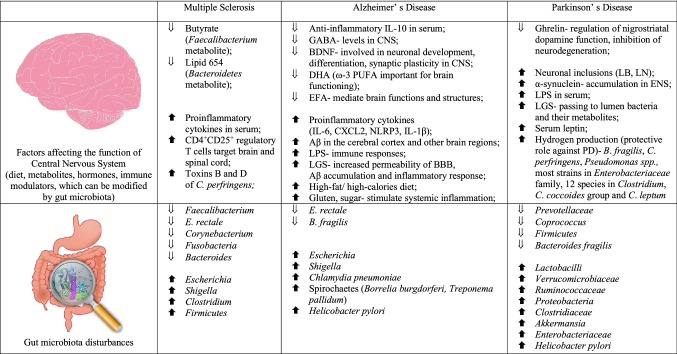
Factors affecting the function of Central Nervous System (diet, metabolites, hormones, immune modulators, which can be modified by gut microbiota) and gut microbiota disturbances in multiple sclerosis, Alzheimer’s disease, Parkinson’s disease

*GABA* gamma-aminobutyric acid, *BDNF* brain-derived neurotrophic factor, *DHA* docosahexaenoic acid, *ω-3 PUFA* omega 3 polyunsaturated fatty acids, *EFA* essential fatty acids, *Aβ* amyloid-β, *LPS* lipopolysaccharides, *LGS* leaky gut syndrome; *CNS* central nervous system, *LB* Lewy bodies, *LN* Lewy neuritis, *ENS* enteric nervous system, *PD* Parkinson’s disease
